# Characteristics of primary health care units with focus on drug information from the pharmaceutical industry and adherence to prescribing objectives: a cross-sectional study

**DOI:** 10.1186/1472-6904-10-4

**Published:** 2010-02-15

**Authors:** Daniel Carlzon, Lena Gustafsson, Anna L Eriksson, Karin Rignér, Anders Sundström, Susanna M Wallerstedt

**Affiliations:** 1 Department of Clinical Pharmacology, Sahlgrenska University Hospital, 413 45 Göteborg, Sweden; 2 Göteborg Primary Health Care, Lillhagsparken 6, 422 50 Hisings Backa, Sweden; 3 Centre for Pharmacoepidemiology, Karolinska Institute, 171 77 Stockholm, Sweden

## Abstract

**Background:**

Adherence to prescribing guidelines varies between primary health care units. The aim of the present study was to investigate correlations between characteristics of primary health care units and adherence to prescribing objectives for rational drug use with focus on drug information from the pharmaceutical industry.

**Methods:**

A cross-sectional study was performed in all 25 primary health care units in Göteborg, Sweden. A questionnaire on characteristics of practice settings [(i) size of unit, (ii) profession of head, (iii) use of temporary physicians, (iv) drug information from the pharmaceutical industry, (v) producer-independent drug information, and (vi) education on prescribing for newly employed physicians] was sent to the heads of the units. A national sales register for prescribed drugs (Xplain) was used for evaluation of adherence to the six regional prescribing objectives concerning proton pump inhibitors (PPIs), angiotensin converting enzyme inhibitors (ACEIs), statins and antidepressants.

**Results:**

Twenty-two out of 25 primary health care units responded to the questionnaire (response rate 88%). A physician as head and presence of producer-independent drug information was positively correlated with adherence to the prescribing objectives (median number of prescribing objectives adhered to (25^th ^- 75^th ^percentile): 2.5 (1-3.25) vs 1 (0-2), P = 0.013; 2 (1-3) vs 0, P = 0.043, respectively. Presence of drug information from the pharmaceutical industry and education on prescribing for newly employed physicians was negatively associated with adherence to the prescribing objectives: 1 (0-2) vs 3.5 (2.25-4.75), P = 0.005; 1 (0-2) vs 3 (1.5-4), P = 0.034, respectively.

**Conclusion:**

Several characteristics of the primary health care units correlated with adherence to prescribing objectives for rational drug use. Further research on this topic is needed and would constitute valuable information for health care decision makers.

## Background

The prescribing of drugs is an important issue for the individual patient, since risks and benefits of the treatment directly affect the patient. In Sweden, prescribed drugs are reimbursed by the society. Hence, prescribing of drugs is also a key question from a public expense perspective. Financing of drugs is a vast problem, since costs for drugs are increasing and resources are limited [1]. Evaluation of costs and benefits for alternative treatment strategies is essential and rational drug use implies physicians' prescribing of drugs with favourable cost-benefit balances.

Guidelines for recommended drugs are important for rational drug use. However, prescribing and adherence to prescribing guidelines vary between health care units [2], for example according to patient characteristics [3-5], physician characteristics [4], practice settings [4], budgetary policies [6] and country of residence [7]. Sources of drug information used by the physicians may be of additional significance [8].

In the region of Västra Götaland, Sweden, prescribing objectives are set up for rational drug use. A national sales register for prescribed drugs (Xplain) is used for evaluation of the objectives. The register includes aggregated data on age, sex, and residential area of the patient, as well as information on the unit from which a drug is prescribed, and the drug dispensed [e.g. number of defined daily doses (DDD) and costs]. Each primary health care unit is responsible for its own budget, which shall cover its expenses for society reimbursements for drugs.

The public primary health care of Göteborg in the region of Västra Götaland, Sweden, consisted in 2007 of 25 units. The head of each primary health care unit receives a monthly report on their results on the prescribing objectives, as well as the overall results of all units in Göteborg. Moreover, pharmacists present the results to the physicians working in the unit at seminars twice a year. Knowledge on factors correlated with adherence to the prescribing objectives is essential for rational use of drug.

The aim of the present study was to investigate correlations between characteristics of primary health care units and adherence to prescribing objectives for rational drug use with focus on drug information from the pharmaceutical industry.

## Methods

A cross-sectional study was performed in all publicly run primary health care units in Göteborg, Sweden (n = 25). A questionnaire including questions on characteristics of practice settings was sent in print by mail to the heads of the primary health care units in November 2007. A letter revealing the source of the questionnaire and assuring anonymity of the units at presentation of results accompanied the questionnaire. Characteristics included in the questionnaire were (i) size of the primary care unit (1 = serving <5000 inhabitants, 2 = 5000-10000 inhabitants, 3 = 10000-15000 inhabitants, 4 = 15000-20000 inhabitants, 5 = >20000 inhabitants), (ii) profession of the head of the unit, (iii) use of temporary physician from other employers, (iv) drug information from the pharmaceutical industry, (v) producer-independent drug information, and (vi) education on prescribing for newly employed physicians. The questionnaire (in Swedish) can be obtained from the corresponding author. Two weeks after sending the questionnaire, an e-mail reminder was sent to those who had not responded.

For the primary health care units responding to the questionnaire, a national register for sales of prescribed drugs (Xplain) was used to extract prescribing data from January-December 2007 concerning results on the six regional prescribing objectives, including prescribing of PPIs (proportion of generic omeprazol), ACEIs (proportion ACEI of ACEIs and angiotensin receptor blockers [ARBs]), statins (proportion of generic simvastatin), and antidepressants (proportion selective serotonin reuptake inhibitors [SSRI] of antidepressants, proportion non-escitalopram of SSRIs and proportion generic mirtazapin of mirtazapins). The regional primary health care authorities arbitrarily defined appropriate adherence level to the prescribing objectives. During 2007 these levels were set at >80%, >70%, >80%, 75%, 95% and 98% of the prescribed and dispensed DDD, respectively.

### Statistics

Statistical analyses were conducted using SPSS 12.0.1. Due to the small number of primary health care units in the study, multivariate logistic regression could not be performed. Spearman correlation coefficients were calculated to evaluate bivariate correlations between questionnaire results and number of prescribing objectives adhered to. Mann-Whitney's test was used for comparison of results on the regional prescribing objectives in primary health care units with or without drug information from the pharmaceutical industry. Fisher's exact test was used for comparisons between categorical values. A P-value < 0.05 was considered significant. Values are presented as median (25^th^-75^th ^percentile) if not stated otherwise. Percentages are calculated with the total number of responding units to the particular question as denominator.

## Results

Twenty-two out of 25 heads of primary health care units responded to the questionnaire (response rate 88%), though all questions were not responded upon. Characteristics of the units according to the questionnaire are presented in Table 1.

**Table 1:** Characteristics of the primary health care units according to the questionnaire (n = 22).

		Number of units (%)	Number of not responding units
Physician as head of unit		10 (48)	1
Size of the unit (number of patients served)	<5000	1 (5)	0
	5000-10000	2 (9)	
	10000-15000	4 (18)	
	15000-20000	9 (41)	
	>20000	6 (27)	
Temporary physicians		12 (55)	0
Drug information from the pharmaceutical industry		17 (81)	1
Producer-independent drug information		19 (86)	0
Education on prescribing for newly employed physicians		16 (76)	1

Values are presented as n (% of the responders).

Bivariate correlations between characteristics of the primary health care units and adherence to the prescribing objectives are presented in Table 2. A physician as head and presence of producer-independent drug information was positively correlated with adherence to the prescribing objectives. Presence of drug information from the pharmaceutical industry and education on prescribing for newly employed physicians was negatively correlated with adherence to the prescribing objectives.

**Table 2:** Correlation between number of prescribing objectives adhered to and factors investigated in the questionnaire

	Number of prescribing objectives adhered to median (25^th^-75^th ^percentile)		P-value
	Yes	No	
Size of the unit	NA	NA	0.14
Physician as head of the unit	2.5 (1-3.25)	1 (0-2)	0.013
Temporary physicians	1 (0-2)	2 (1-3)	0.051
Drug information from the pharmaceutical industry	1 (0-2)	3.5 (2.25-4.75)	0.005
Producer-independent drug information	2 (1-3)	0	0.043
Education on prescribing for newly employed physicians	1 (0-2)	3 (1.5-4)	0.034

The total of six prescribing objectives concerned PPIs (proportion of generic omeprazol), ACEIs (proportion ACEI of ACEIs and angiotensin receptor blockers [ARBs]), statins (proportion of generic simvastatin), and antidepressants (proportion selective serotonin reuptake inhibitors [SSRI] of antidepressants, proportion non-escitalopram of SSRIs and proportion generic mirtazapin of mirtazapins). The arbitrarily defined appropriate adherence levels to the prescribing objectives were set to 80%, 70%, 80%, 75%, 95% and 98% of the prescribed and dispensed DDDs, respectively.

ACEI, angiotensin converting enzyme inhibitor; ARB, angiotensin receptor blocker; DDD, defined daily dose; NA, not applicable; PPI, proton pump inhibitor; SSRI, selective serotonin reuptake inhibitor

Seventeen out of the 21 responding units (81%) received drug information from the pharmaceutical industry (one of which did not respond to the question on profession of the head of unit). Presence of drug information from the pharmaceutical industry was less common with physician as head of the unit; six of ten units headed by a physician had received information from the pharmaceutical industry, whilst all ten units headed by another profession had done so (P = 0.087). Presence of drug information from the pharmaceutical industry was more common in units with education on prescribing for newly employed physicians;15 out of 17 units receiving drug information from the pharmaceutical industry had education on prescribing for newly employed physicians, whilst the corresponding number for units without drug information from the pharmaceutical industry was 1 out of 3 (P = 0.088). No other correlations with P < 0.10 were found between characteristics of the units revealed in the questionnaire. Units with and without visits from the pharmaceutical industry were geographically spread in the area.

Characteristics of the drug information from the pharmaceutical industry are presented in Table 3. In 14 out of 17 units (82%), representatives from other professions than physicians also attended the information sessions. In 8 units (47%), information was given more than once a month.

**Table 3:** Characteristics of drug information from the pharmaceutical industry in units receiving this kind of information (n = 17)

		Number of primary health care units n (%)
Frequency of visits	Once a week	4 (24)
	>Once a month	4 (24)
	<Once a month	9 (53)
Attending professions	Physicians only	3 (18)
	Physicians and nurses	5 (29)
	All personnel	9 (53)
Time of information	Lunch ≥50% of occasions	13 (81)
	Working hours ≤50% of occasions	14 (88)

The results on the six regional prescribing objectives in primary health care units with or without drug information from the pharmaceutical industry are presented in Table 4. In 2007, the median number of DDD per health care unit with and without drug information from the pharmaceutical industry was for PPIs 88 836 (60 995-193 230) and 58 921 (40 097-72 843); for ACEIs and ARBs 201 186 (159 657-319 914) and 139 755 (92 952-199 299); for statins 179 632 (144 807-248 018) and 143 463 (100 468-222 974); for antidepressants 185 875 (143 846-251 642) and 127 308 (75 052-179 393); for SSRIs 136 127 (107 924-172 396) and 95 649 (58 281-134 935); and for mirtazapins 26 346 (20 774-31 695) and 12 469 (7 224-19 577).

**Table 4:** Results on the regional prescribing objectives in primary health care units with or without drug information from the pharmaceutical industry.

	Without drug information from the pharmaceutical industry (n = 4)	With drug information from the pharmaceutical industry (n = 17)	P-value*
Generic omeprazole of PPIs	77 (73-89)	77 (72-81)	0.59
ACEIs of ACEIs and ARBs	62 (52-72)	59 (54-61)	0.72
Generic simvastatin of statins	84 (81-92)	75 (71-80)	0.016
SSRI of antidepressants	77 (74-78)	71 (69-74)	0.020
Non-escitalopram of SSRIs	96 (94-97)	94 (91-96)	0.21
Generic mirtazapin of mirtazapins	84 (78-93)	85 (77-91)	0.86

Values are presented as median percentage (25^th^-75^th ^percentile) of prescribed DDDs.

ACEI, angiotensin converting enzyme inhibitor; ARB, angiotensin receptor blocker; DDD, defined daily dose; PPI, proton pump inhibitor; SSRI, selective serotonin reuptake inhibitor

*Mann Whitney's test

## Discussion

The results of the present study indicate that the profession of the head and the source of drug information may be of importance for rational prescribing, as measured by adherence to prescribing objectives. Some of these factors are correlated with each other, and a study including more health care units would be of interest to identify independent factors for adherence to prescribing objectives. Therapeutic traditions at health care units have previously been shown to influence prescribing [9].

The head of a health care unit has the responsibility for the operational activity. Hence, this post is of utmost overall importance. However, to the best of our knowledge the importance of this post for the prescribing of drugs has not been investigated before.

Interestingly, for all health care units not receiving drug information from the pharmaceutical industry, the head of the unit was a physician. For the remaining units, the head was a physician in only 6 of 16 cases (38%). From our experience, representatives from the pharmaceutical industry invite themselves for drug information sessions rather than are invited by health care representatives. Hence, it is up to the head to decide whether to accept or reject the offer. The present study does not allow conclusions concerning rationales for the heads' choice concerning this matter. However, non-physician heads may have less knowledge on what sort of information is provided. Their understanding of the prescribing process may also be less prominent since prescribing is not their area of expertise as they do not prescribe themselves. The rationales for heads to accept/reject drug information from the pharmaceutical industry would be of interest to investigate further.

The majority of primary health care units received drug information from the pharmaceutical industry, predominantly at lunchtime which is not included in working hours in Sweden. In many cases, representatives from professions without prescribing authority, but who work in close contact with patients, also receive the information. Whether these groups of professionals may influence patients' requests of certain drugs is not known.

In the present study, source of drug information was correlated with prescribing. Primary health care units not receiving drug information from the pharmaceutical industry succeeded in adhering to the majority of the prescribing objectives for rational drug use, whereas those receiving information adhered to a far less extent. Our results confirm the results of other studies, where visits by pharmaceutical sales representatives were associated with a broader range of drugs prescribed [10] and increased prescribing costs [11,12].

Drug information is important for rational drug use, which requires adequate knowledge on drugs' benefits, risks and cost-effectiveness. The increasing volume of information related to drugs and prescribing may make it difficult for an individual primary care physician to keep up to date with best practice. In 2007, all primary health care units in Göteborg were offered producer-independent drug information sessions from the Department of Clinical Pharmacology. An important finding in the present study is the correlation between producer-independent drug information and rational drug use. The majority of the health care units received this kind of information and it would be of interest to learn more about the rationales for the rejection of this offer. Producer-independent drug information has previously been shown favorable for rational prescribing [8].

A finding worth further attention is that education of newly employed physicians on prescribing was negatively correlated with rational drug use. This raises the question on the quality of this education. Our results also raise the question on the content and quality of drug information from the pharmaceutical industry. Is it possible to distinguish marketing and information from a source dependent on sales of the products about which they inform? The influence from the pharmaceutical industry on prescribing is also of interest from another point of view, since it is a distinct external entity separate from the health care and therefore possibly more easily controlled than factors ascending from the health care itself, i.e. we can decide whether to receive it or not.

A limitation of the present study is the small sample size of 25 health care units, which makes multivariate analyses inappropriate. Nevertheless, the study showed several statistically significant findings, which would be worthwhile to investigate further.

## Conclusion

Profession of head and sources of drug information correlate with adherence to prescribing objectives for rational drug use. Further research on this topic is needed and would constitute valuable information for health care decision makers.

## Competing interests

The authors declare that they have no competing interests.

## Authors' contributions

DC participated in the design of the study, drafted the questionnaire, carried out the acquisition of data, drafted and revised the manuscript. LG participated in the design of the study, revised the questionnaire, carried out the acquisition of data and revised the manuscript. ALE revised the questionnaire and revised the manuscript. KR participated in the design of the study, revised the questionnaire and revised the manuscript. AS contributed to the statistical analyses and revised the manuscript. SMW conceived the study, participated in its design, revised the questionnaire, performed the statistical analyses, drafted and revised the manuscript. All authors read and approved the final manuscript.

## Pre-publication history

The pre-publication history for this paper can be accessed here:

http://www.biomedcentral.com/1472-6904/10/4/prepub
